# Study of the Effects of Aging Treatment on Astroloy Processed via Hot Isostatic Pressing

**DOI:** 10.3390/ma12091517

**Published:** 2019-05-09

**Authors:** Emilio Bassini, Giulio Cattano, Giulio Marchese, Sara Biamino, Daniele Ugues, Mariangela Lombardi, Gianfranco Vallillo, Benjamin Picqué

**Affiliations:** 1 Politecnico di Torino, DISAT: Department of Applied Science and Technology, Corso Duca degli Abruzzi, 24, 10129 Torino, Italy; giulio.marchese@polito.it (G.M.); sara.biamino@polito.it (S.B.); daniele.ugues@polito.it (D.U.); Mariangela.lombardi@polito.it (M.L.); 2 Center for Sustainable Futures @ Polito, Istituto Italiano di Tecnologia, Corso Trento 21, 10129 Turin, Italy; giulio.cattano@polito.it; 3 Consorzio Interuniversitario Nazionale per la Scienza e Tecnologia dei Materiali (INSTM), Via G. Giusti, 9, 50121 Firenze, Italy; 4 Avio Aero, Engineering Materials and Processes, Via I Maggio, 99 10040 Rivalta di Torino (TO), Italy; Gianfranco.Vallillo@avioaero.it; 5 AUBERT & DUVAL–Pamiers Business & Development manager NNS & NS Parts Powder Metallurgy 75, bd de la Libération-B.P. 173, 09102 Pamiers CEDEX, France; benjamin.picque@eramet-aubertduval.com

**Keywords:** astroloy, hot isostatic pressing, ageing heat treatment, metals and alloys, powder metallurgy, grain boundaries, high-temperature alloys

## Abstract

The effect of aging treatment on Astroloy fabricated via hot isostatic pressing and subjected to super-, and sub- solvus solutioning has been investigated. The evolution of hardness and microstructural features were followed after each step of the treatment. Since this alloy is commonly subjected to a double aging treatment at two different temperatures, particular attention was given to the effectiveness of the first aging treatment compared to the second one. Coarsening and modification of the γ′ reinforcing system together with carbides formation were made the object of research. The cooling rate used after solutioning treatment was also kept into account. Finally, a model to describe secondary and ternary gamma prime coarsening upon aging treatments is presented.

## 1. Introduction

Since aeronautical engine efficiency is tightly linked with the operative temperature, turbine designer and manufacturers continuously aim at elevating this functioning parameter. To accomplish this task, traditional alloys must be substituted to withstand such a demanding environment. The most suitable material category to realize forged aeronautical components for such high operative temperature is nickel-based superalloy. These alloys are typically reinforced through precipitation hardening which strongly relies on the γ′ phase (i.e., Ni_3_(Al, Ti) intermetallic volume fraction achieved upon heat treatment. By modifying the alloys’ composition, it is possible to increase γ′ precipitation, sometimes reaching a precipitates volume fraction of 60%. To obtain this result, more Ti and Al are added to the alloy together with other refractory elements which reinforce the matrix mainly by carbides formation, i.e., Mo, W, Ta, and Hf. However, these modifications lead to strong segregation in the cast ingots and to a dramatic reduction in formability by forging. According to this, nowadays, a powder metallurgy route called near net shape hot isostatic pressing or (HIPping) is considered very promising to manufacture parts made of those Ni superalloys that are difficult to forge [1,2,3,4,5,6,7,8,9,10,11,12,13,14,15,16,17]. This procedure offers a reduction of machining operations and of raw material consumption concerning forging with a consequent reduction of buy to fly ratio as demonstrated by Bassini et al. in [18].

Nevertheless, this process must be carefully optimized to reduce the detrimental effects correlated to the prior particle boundaries (PPB) occurrence. Wanhang and Waters in their works [19,20] assessed the impact on mechanical properties of PPBs in as-hipped and in hipped and heat-treated samples. Furthermore, a HIP processing step alone is not enough to ensure mechanical properties compliant with aeronautical requirements thus a post-heat treatment is mandatory, as demonstrated by Roncery in his work [21] about SX nickel alloy obtained via HIPping. Similarly to forging, it is necessary to subject components to a complete heat treatment which consists of a solutioning followed by aging.

Astroloy is one of these Ni superalloys with high γ′ content and, consequently, better mechanical properties, but lower forging capability. This material can take clear advantage by HIPping. According to the literature [7,22,23,24,25,26,27,28,29,30], for precipitation-hardened superalloy such as Inconel 718, Inconel 625, FGH 4096, Waspaloy and Astroloy, microstructural features like γ′ size, volume fraction and dispersion greatly affect material properties. For example, the work of Rao on Inconel 718 [7] evidenced the relationship between the size of the reinforcing particles and the final mechanical properties of the samples. Similarly, Fu, who worked on a γ′ reinforced alloy similar to Astroloy, found a relationship between the size and shape of the particles and the workability of the material [22]. Clearly, all these γ′ properties can be strongly altered varying heat treatment parameters as indicated in the published work of Balakrishna [30]. For each alloy and specific starting metallurgical state, it is necessary to establish solid knowledge of the fine control of microstructural evolution upon thermal history.

In previous work from Bassini et al. [8], the solutioning step of heat treatment was already optimized. In the current study, the optimization of the aging step is presented. The aging step has deep effects on γ′ and carbides evolution [31,32,33,34,35,36,37,38,39]. For example, Dong in his work [31] focused on the effects brought by the formation of carbides at grain boundaries in a Ni-based superalloys pointing out their embrittlement effects. Other researchers like Zhang accurately examined the evolution of the carbides during the ageing treatment in FGH96 Ni-based superalloys [32]. On the other hand, Collins examined the MC carbides stability and their eutectic transformation into M_23_C_6_ + γ′ [33] in a Ni-Co-Cr superalloy. The present work assesses the formation and growth of γ′, intragranular and intergranular carbides, which are typically in the MC, M_6_C and M_23_C_6_ form.

Wrought Astroloy is typically heat treated following a complex recipe including a high-temperature solutioning step followed by a stabilizing step at 980 °C and finally, a two-step aging. The stabilizing step is used to promote the transformation of MC carbides in more stable M_23_C_6_ carbides at grain boundaries (GBs)

M_23_C_6_ carbides have a significant effect on the mechanical properties of superalloys. They form discontinuous blocky grain-boundary precipitates, and they are mainly beneficial in this form because they prevent grain-boundary sliding at high temperature. A higher fraction of M_23_C_6_ carbides at GBs increase serration effects which are known to decrease the crack propagation rate. Despite this, It should be recalled, however, that even as the blocky grain-boundary carbide they limit ductility by either fracturing or decohesion of the carbide-matrix interface [40]. Gessinger, however, reviewed that the application of the stabilizing process to Astroloy obtained via HIP decreases the mechanical properties at 620 °C and creep life. Similar conclusions were drawn by Gayda and Brunetaud in their work on Astroloy [41,42]. For these reasons, the stabilizing step was excluded from the optimization process performed within this research work. Starting from a reference heat treatment published in the work of Raisson [1] for powder metallurgy Astroloy, which suggest the first aging at 760 for 8 h and a second aging at 650 °C for 24 h, this work investigated in detail a potential optimization of such heat treatment. In particular, the effects of aging treatment on Astroloy parts obtained by HIPping was studied. Evolution of γ′ precipitates regarding volume fraction and the average size has been correlated to micro-Vickers hardness levels. Furthermore, a study about the evolution of carbides upon heat treatment has been performed.

## 2. Materials and Methods

Commercial Astroloy powders were consolidated via hot isostatic pressing in the shape of a 100 mm × 100 mm × 1000 mm bar. The chemical composition of this alloy is reported in Table 1. Chemical composition was obtained via plasma emission spectrometry. Carbon and sulfur were revealed through combustion. Equipment used was an atomic emission spectrometer model TJA Iris Advantage. Oxygen and Nitrogen levels were assessed with melting under inert gas using a LECO TC436AR analyzer (Leco, St. Joseph, MI, USA).

HIPping was performed above γ′ solvus range, i.e., above the range 1115–1130 °C, to reduce the influence of PPB and to promote grain recrystallization across particles as demonstrated by [1]. Each heat treatment recipe was applied on a set of five Astroloy cubes with 100 mm sides. These test coupons were removed by cold cutting from the hipped bar. More precisely, the HIP cycle was performed at 1160 °C with a soaking time of 4 h applying 100 MPa; the pressure and the temperature were increased and decreased at the same time.

### 2.1. Sample Pre-Treatment

**Figure 1:**
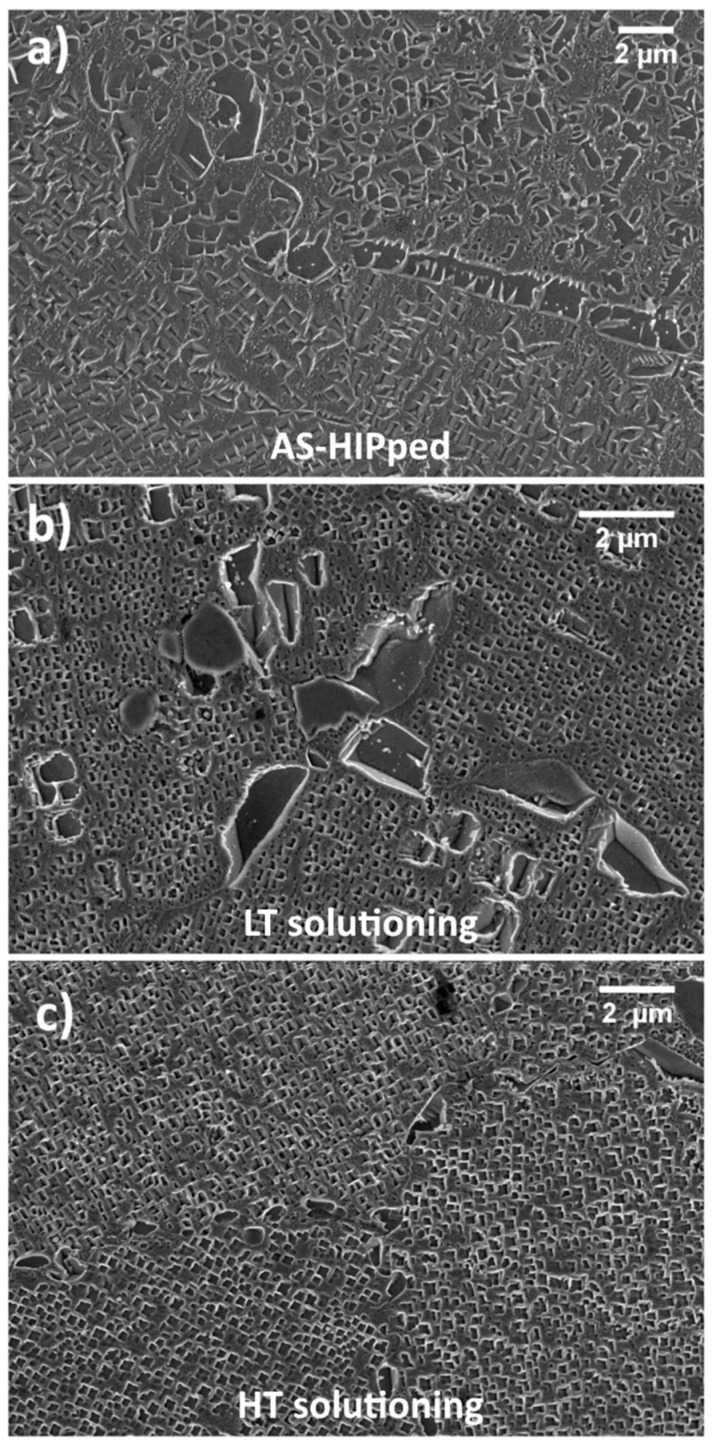
Astroloy in the as-HIPped (hot isostatic pressing) condition (**a**); after sub-solvus solutioning (**b**); after super-solvus solutioning (**c**).

As-Hipped samples, which presented the coarse microstructure evidenced in Figure 1a were divided into two sets and subjected to solutioning at two different temperatures using a vacuum furnace. A Sub-solvus treatment, 1115 °C, and a super-solvus one, 1160 °C, were performed. The same soaking time of 4 h was used independently by the solutioning temperature used. In previous work, such solutioning conditions named low temperature (LT) and high temperature (HT) were found to be the most promising recipes for this alloy [8,37].

HT and LT samples were cooled to room temperature after solutioning at 70 °C/min by modulating the Nitrogen pressure into the furnace during the cooling stage. Depending on the selected solutioning temperature, the two following structures can be achieved as presented in Figure 1b,c.

This step of the heat treatment was performed into a TAV MINIJET HP S/N 235 horizontal vacuum furnace (TAV, Caravaggio, Bergamo, Italy) where pressure was maintained at 10^−2^ mbar. These treatment conditions are summarized in Table 2. After these treatments, γ′ amount was assessed to be 44% and 35% in volume for LT and HT samples respectively as also shown in [8]. The major difference observed between the two solutioning conditions is the achievement either of a partial (LT) or an almost complete (HT) dissolution of primary γ′ at GBs. Therefore, the metallurgical state of LT and HT samples before aging steps exhibits a bimodal size distribution and a uniform size distribution of γ′, respectively.

### 2.2. First and Second Ageing

Since this work deals with the effect of aging treatments on the alloy microstructural features, different aging temperatures and times were investigated on samples solutioned as earlier described. As far as the first aging is concerned, a set of time and temperature levels were applied to identify the optimal combination of these parameters. The optimization criterion was the assessment of hardness evolution and, in particular, the localization an aging peak hardness (PH). Once, the optimal first aging recipe was defined, optimization trials on second aging steps were carried out.

The temperature ranges in which performing the optimization of the aging treatments were first roughly identified with Differential Scanning Calorimetry (DSC) analysis.

DSC tests were performed using a Setaram TGA-DSC 9216.18 (Setaram, Caluire, France) applying a heating rate of 20 °C/min in the temperature range between 25 and 850 °C. Tested samples had a diameter and a height of 3 mm and 10 mm respectively with a weight of 180 mg. The DSC analysis was performed on one as-hipped sample and two solutioned samples obtained with the same parameters described in Figure 3.

Following the DSC outcome, the first aging heat treatment trials were performed at two different temperatures, i.e., 760 and 785 °C while the soaking time was varied from 6 to 24 h. The second aging trials were performed at a lower temperature, i.e., 650 and 675 °C with soaking time ranging from 20 to 48 h. These treatments were performed in a static atmospheric furnace Nabertherm LH 60/14 (Nabertherm Gmbh, Lilienthal, Germany) and samples were air cooled outside the furnace.

### 2.3. Observation of the Microstructure

To observe the final microstructure achieved after the heat treatment steps, each sample was ground up to 2400 grid papers and then polished with diamond pastes down to 1 μm. Metallography samples were chemically etched with waterless Kalling’s whose composition is 2.5 g CuCl_2_ + 50 mL HCl + 50 mL EtOH. Short etching time, lower than 45 s, were used to study γ′ precipitates. In a separate test, longer etching times, typically 180 s, were applied to properly evidence grains structure whose dimension was calculated using the intercept methods as reported in ASTM E112 standard.

**Figure 2:**
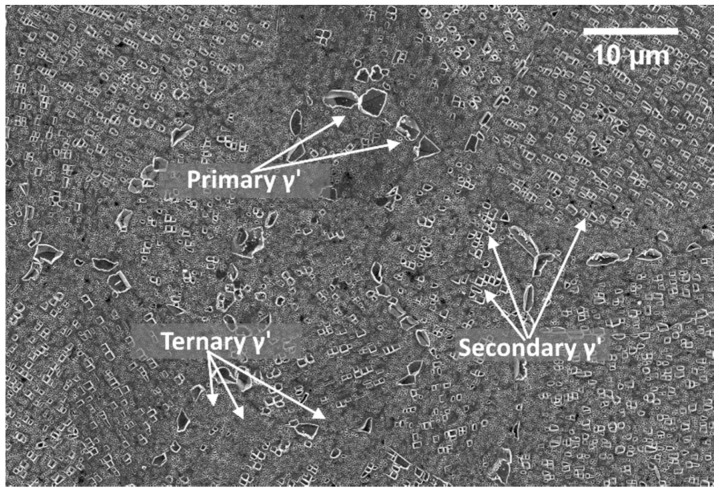
Generic micrograph showing the three γ′ categories, i.e., primary, secondary and ternary.

The images representing the average microstructure and grains dimension were obtained through optical microscope Leica MEF4M (Leica, Wetzlar, Germany), while γ′ analysis was performed with a field emission high-resolution scanning electron microscope (FESEM, Zeiss Merlin, (Carl Zeiss, Oberkochen, Germany) equipped with a Gemini column. An Electron Dispersive X-Ray Spectroscopy EDS probe was used to gather compositional information about carbides which were observable using SEM backscattered electron detector of an SEM LEO 1450 VP. Volume fractions of γ′ populations, and their related size was derived by treating through image processing 10 FESEM images taken at 20kX magnification. A careful image selection was made to consider portions of the microstructure where only square secondary and tertiary γ′ features were observable. Image processing was done via ImageJ software (National Institutes of Health, Bethesda, MD, USA). Image contrast was enhanced to reveal the intermetallics. Once they were clearly distinguishable from the matrix, ImageJ was used to create a binary image (black and white image) in which the black portion represents the γ′ phase. The software assesses the area of the portions mentioned above and the ratio with the entire picture area is calculated. This ratio equals the volume fraction of particles since a unity thickness is concerned. Intermetallic precipitates within Astroloy can be divided into three categories according to their size, i.e., primary ($\gamma_{p}^{\prime }$), secondary ($\gamma_{s}^{\prime }$) and ternary ($\gamma_{t}^{\prime }$) as depicted in Figure 2.

For the assessment of the precipitate populations, the following conventions were adopted: except for $\gamma_{p}^{\prime }$ that has irregular shape γ′ particles are considered cubic, therefore edges were used to categorize their size. To account for their irregular shape, $\gamma_{p}^{\prime }$ particle sizes have been calculated using the equivalent ellipses treatment. In particular, particles with cubic edges up to 300 nm were classified as $\gamma_{t}^{\prime }$, 300 and 700 nm were classified as $\gamma_{s}^{\prime }$ while particles with an aspect ratio greater than 5, and with a major axis larger than 800 nm were classified as $\gamma_{p}^{\prime }$. Carbides size and volume fraction were calculated in the same manner as the intermetallic particles using ImageJ. In this specific case carbides, were identified thanks to their intense white color under backscattered electrons. Since they are mostly spherical, equivalent ellipses were used to measure their size and geometrical characteristics. Hardness measurements were performed using a Vickers micro-hardness test, averaging five indentations per sample. This test was used on samples applying a load of 500 g for 15 s. The equipment used was a Leica Microvickers VMHT. Finally, X-ray diffraction (XRD) analysis were performed with an X-Pert Philips diffractometer (PANalytical, Almelo, The Netherlands) by CuKα radiation in a Bragg Brentano configuration working at 40 KV and 40 mA with a step size of 0.013°. The XRD diffractogram was recorded from 30° to 100°. Samples were analyzed in the as-hip condition, after solution treatment and after receiving the first and second ageing treatment respectively but only in the peak-hardness condition.

## 3. Results

### 3.1. Differential Scanning Calorimetry (DSC) Results

**Figure 3:**
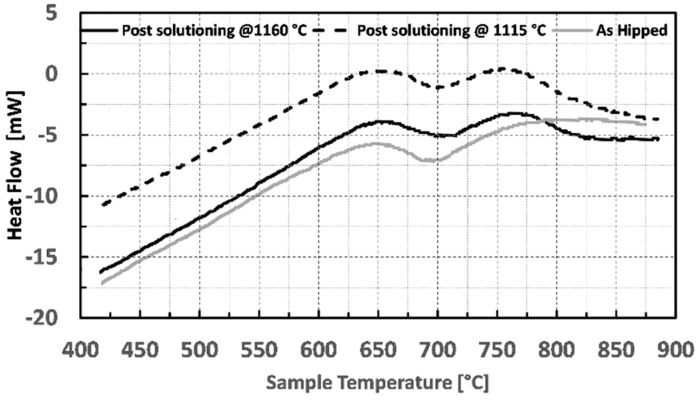
Differential Scanning Calorimetry (DSC) analysis for Astroloy in the as HIPped condition (gray line), after solutioning at 1160 °C (solid black line) and after solutioning at 1115 °C (dotted black line).

Figure 3 shows the obtained heat flow as a function of the temperature of the samples.

The two solutioned samples clearly show two distinct exothermic peaks centered at 650 and 760 °C, even though with different enthalpies. Peaks for the HT samples are stronger concerning those of the LT samples indicating a greater γ′ precipitation. This behavior is due to the high amount of γ′ dissolved with HT solutioning, resulting in a γ matrix richer in γ′ former elements, thus promoting more intense precipitation of such a phase. In particular, the enthalpies obtained for the first and second peak were −0.56 and −0.29 J for the HT sample and −0.47 and 0.17 J for the LT respectively. On the other hand, the as-hipped sample shows only one peak at 650 °C (−0.52 J), while the second is particularly broad, and it is not completely observable in the studied temperature range. Therefore, the enthalpy of this second peak was not calculated on the as-HIP sample. The position of the peaks is in good agreement with the aging temperatures found in the literature for the wrought alloys [1], thus confirming that the temperature ranges studied in this paper are the most suitable to reinforce the HIPped alloy via precipitation hardening.

### 3.2. First Ageing γ′ Assessment

**Figure 4:**
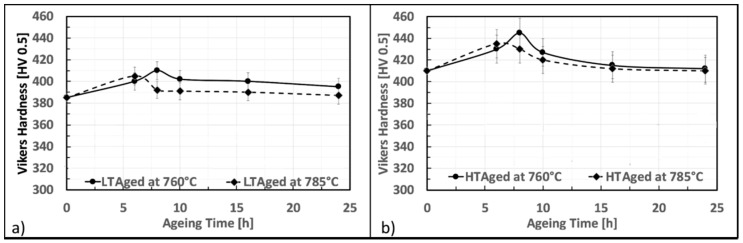
First aging curves for low-temperature (LT) samples (**a**) and high-temperature (HT) samples (**b**).

First aging steps were carried out at a higher temperature concerning the second one. This strategy was adopted since it was thought that a higher temperature during the first aging would have increased the number of nucleation sites, and thus, the final volume fraction of precipitates. The second aging steps, on the other hand, were carried out at a lower temperature to promote a further fine γ′ precipitation, limiting the coarsening of the former intermetallics, already formed during previous stages. Figure 4a,b show the first aging effect on LT and HT solutioned samples. Specimens were aged at 760 and 785 °C and are described by hardness aging curves similar in shape. Material strengthening occurs between the first 6 or 8 h. Longer soaking time causes a progressive alloy softening. When a higher aging temperature is employed, i.e., 785 °C, peak hardness is reached in a shorter time, i.e., 6 h. On the other hand, when aging is performed at 760 °C, peak hardness appears after 8 h. Independently by the solutioning recipe adopted, aging at 760 °C always resulted in a higher peak hardness concerning aging at 785 °C. Hardness increase at peak conditions is greater for HT samples and, in particular, after aging at 760 °C for 8 h an increase of 35 HV is registered. The same aging treatment applied to the LT sample leads to peak hardness, even if the hardness increase is smaller, i.e., only +20 HV.

**Figure 5:**
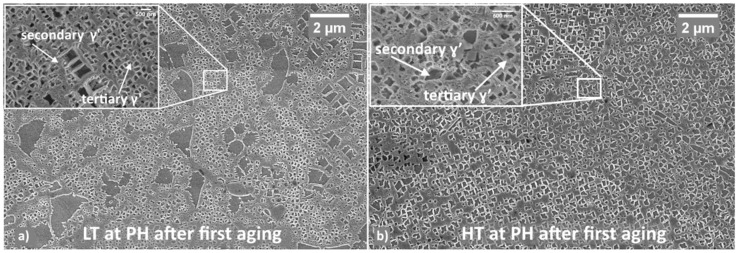
LT sample after aging at 760 °C for 8 h (**a**); HT sample aged at 760 °C for 8 h (**b**).

Based on the above results, most promising samples are LT and HT both first aged for 8 h at 760 °C. Figure 5 shows the microstructures of these two samples as observed through the FESEM imaging.

From the comparison of these structures with those observed in the as-solutioned state (Figure 1b,c), it appears that additional fine γ′ has precipitated. As for LT samples, primary γ′ is still visible and is surrounded by secondary γ′. Also, ternary γ′ is present as shown in the inserts. Similar behavior is also noticeable in HT samples even though, primary γ′ is almost disappeared, and secondary γ′ is the predominant reinforcing system. These two conditions, which led to the best properties, i.e., peak hardness, were further subjected to a second aging treatment and results are presented below.

FESEM images have been processed to study the amount and morphology of γ′ precipitates in the peak hardness condition. In the LT sample, overall γ′ volume fraction is 48% vol. Its microstructure appeared heterogeneous and was made up of three main classes: primary, secondary and tertiary respectively. Observation at higher magnifications allowed to identify a fourth class of γ′ which was defined as “ultra-fine tertiary” since it was even smaller than ternary γ′, i.e., <300 nm.

The main features of the different γ′ classes are as follows:

1. $\gamma_{p}^{\prime }$: these are irregular-shaped particles, always located at the γ grain boundaries. They have an elongated shape and, as a consequence, they were treated in terms of equivalent ellipses. The principal axis is typically 1.5 µm long and the aspect ratio is ca. 7. $\gamma_{p}^{\prime }$ accounts for the 24% of the total detected γ′;
2. $\gamma_{s}^{\prime }$: these particles are typically constituted by an array of sharp cuboidal precipitates arranged along preferential directions randomly dispersed in the austenitic grains. $\gamma_{s}^{\prime }$ has a representative size of ca. 606 nm and accounts almost for the 30% of the total γ′;
3. $\gamma_{t}^{\prime }$: these particles are uniformly dispersed throughout the matrix, in the interspace among coarser precipitates. $\gamma_{t}^{\prime }$ has an average size of 230 nm and constitutes the largest part of the total γ′ fraction, i.e., 40%.
4. Ultra-fine $\gamma_{t}^{\prime }$: this type of particles accounts for the 6% of the total γ′ and it has an average size of about 50 nm. It is mainly located around the coarsest precipitates.

In the HT samples, on the other hand, the total γ′ volume fraction was assessed to be 51%, thus slightly higher than in the LT samples.

In this case γ′ populations can be described as follows:

1. $\gamma_{p}^{\prime }$ results much thinner if compared to the former case. Aspect ratio can be as high as 12 with the major axis that ranges between 0.8 and 1.3 µm. It accounts for less than the 2% of the total γ′ vol fraction.
2. $\gamma_{s}^{\prime }$ has an average size of 310 nm and accounts for the 73% of the total γ′
3. $\gamma_{t}^{\prime }$ shows an average size of 213 nm and represent the 25% of the total γ′

When comparing the two HT recipes, precipitates morphology obtained after super-solvus solutioning appear more uniform and finer if compared to the sub-solvus treatment. This fact could explain the higher hardness level reached at the end of aging treatment. On the other hand, the sub-solvus solutioning has provided, after aging, an additional population of very fine γ′ precipitates the presence of which could be beneficial for the part in service.

### 3.3. Second Ageing γ′ Assessment

**Figure 6:**
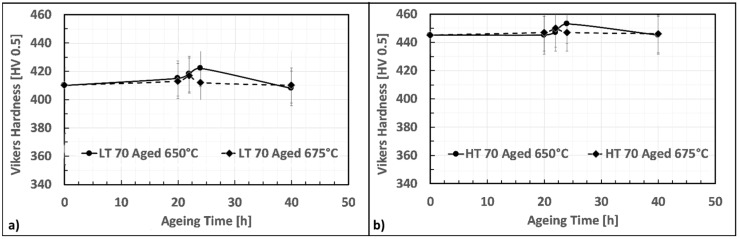
Second aging hardness curve for LT sample (**a**) and HT samples (**b**).

The effects of a further second aging on the material as conditioned as described in the previous section were then investigated. Figure 6 shows the hardness modulation according to time and temperature conditions during this step of the heat treatment. The second aging at 650 °C of LT samples resulted in a slight strengthening of the material with a peak hardness achieved at 24 h. The recorded value at PH is 422 HV, i.e., +12 HV points above the previously achieved hardness level. After this aging time, the material hardness gradually decreases. For LT samples, second aging at 675 °C has also induced an additional strengthening, but properties improvement is lower (i.e., +8 HV). In this case, peak hardness has occurred slightly earlier than 24 h (22 h) and achieved a maximum level of 380 HV. Second aging hardness curves of HT samples are very similar in behavior to those of LT samples, but the overall hardness improvement is smaller (i.e., +10 HV after 24 h at 760 °C and +6 HV after 22 h at 785 °C).

**Figure 7:**
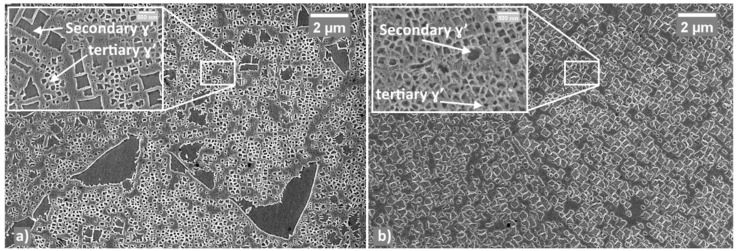
The microstructure of LT sample after second aging (**a**) and HT sample (**b**).

Figure 7 shows microstructures of LT and HT samples, which reached the peak hardness after the second aging.

In LT samples a fine microstructure (as regards $\gamma_{s}^{\prime }$ and $\gamma_{t}^{\prime }$) was detected. Such structure is actually characterized by micron-sized inter-grain γ_p_′ and carbides as well as by sub-micron intra-grain $\gamma_{s}^{\prime }$ and $\gamma_{t}^{\prime }$ particles. An increase of the amount of intra-grain particles can be observed after 24 h. For HT samples, change of γ′ amount appeared very limited with respect to what was observed after the first aging as it will be shown in the next paragraph.

In LT samples the overall γ′ volume fraction has been assessed to be 51% and γ′ particles were classified as follows:

1. $\gamma_{p}^{\prime }$: The principal axis still ranges between 1.5 and 1.6 µm in terms of length and the aspect ratio didn’t change with respect to first aging conditions. $\gamma_{p}^{\prime }$ accounts for the 23% of the total γ′ detected;
2. $\gamma_{s}^{\prime }$ has an average size of ca. 628 nm, and accounts for ca. the 23% of the total γ′;
3. $\gamma_{t}^{\prime }$ has an average size of ca. 237 nm and constitutes the 51% of the total γ′.
4. Ultra-fine $\gamma_{t}^{\prime }$: this type of particles represents the 3% of the total γ′ with an average size of 70 nm.

On the other hand, the microstructure of HT samples, with a total amount of γ′ volume fraction of about 54% can be described as follows:

1. $\gamma_{p}^{\prime }$ exhibits an aspect ratio of 12 with the major axis ranging between 0.8 and 1.4 µm. It accounts for the 1.5% of the total γ′.
2. $\gamma_{s}^{\prime }$ has an average size of 323 nm and accounts for the 62% of the total γ′
3. $\gamma_{t}^{\prime }$ shows an average size of 233 nm and accounts for the 36.5% of the total γ′
4. No trace of ultra-fine $\gamma_{t}^{\prime }$ was detected.

### 3.4. Grain Coarsening

**Figure 8:**
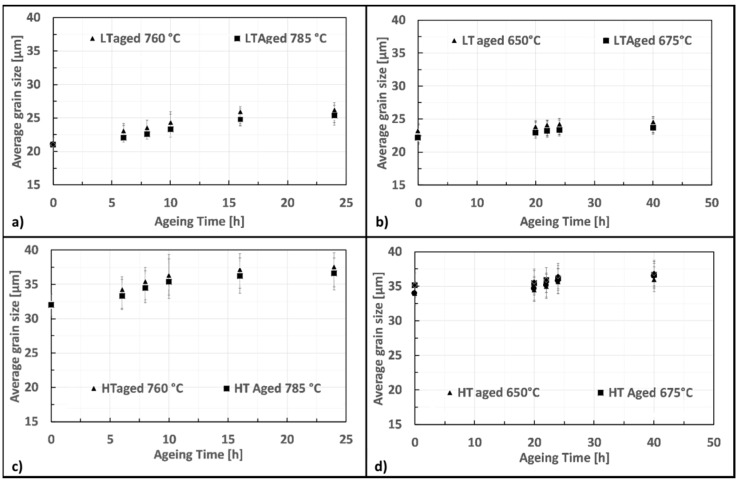
Effect of first and second aging on grain growth for LT and HT specimens: (**a**) first aging on LT, (**b**) second aging on LT, (**c**) first aging on HT and (**d**) second aging on HT.

Figure 8 shows how time and temperature modified the grain size from the as-solutioned condition during first (Figure 8a,c) and second (Figure 8b,d) aging. Grain size is only slightly increased by the first and second aging. In particular, HT samples result to be more susceptible to grain coarsening. However, looking at the results concerning the best first aging recipe (8 h at 760 °C) the grains pass from 21 to 22.5 μm for LT and from 32 to 35 μm for HT, indicating that this effect is minimal and, practically, negligible.

### 3.5. Carbides Assessment

**Figure 9:**
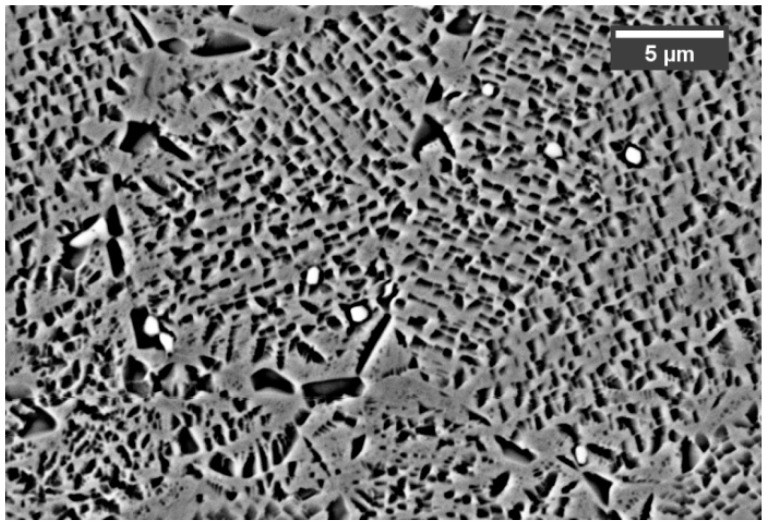
Carbides of as-hipped Astroloy: they appear bright when observed with backscattering detector.

Further to γ′, carbides are very likely to precipitate in Astroloy during HT. The evolution of these particles was studied from the as-hipped state and after each step of the treatment. Carbides form from the HIP consolidation steps, as shown in Figure 9 which refers to the as-hipped state. Here, carbides appear bright and mainly spherical with an average diameter of 625 nm and a volume fraction of 0.36%. They are expected to nucleate and grow both at intra- and inter-grain locations.

**Figure 10:**
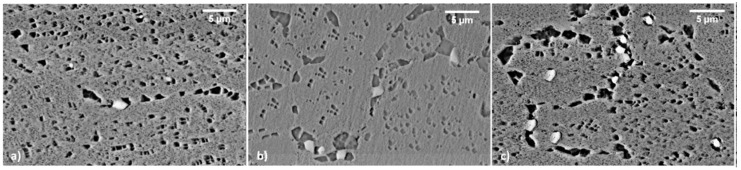
LT samples after solutioning (**a**) after first aging (**b**) and after second aging (**c**).

**Figure 11:**
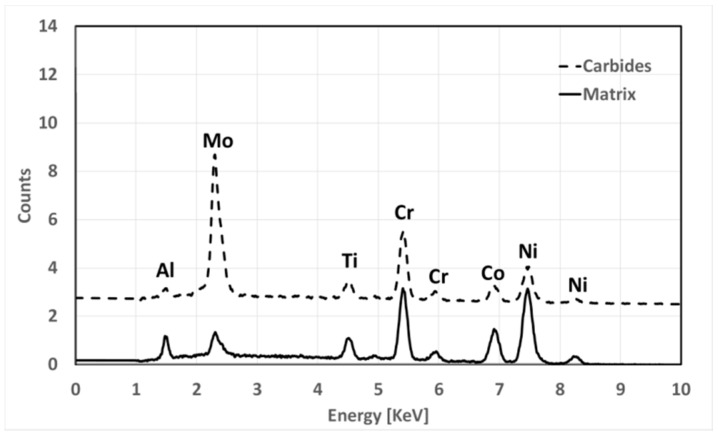
Electron Dispersive X-Ray Spectroscopy (EDS) spectra comparing the matrix (black line) and Mo carbides (dotted line) located at grain boundaries and inside grains.

Figure 10 shows carbides evolution for LT samples in different metallurgical states. Solutioning at 1115 °C does not allow to dissolve them which remain almost unaltered. In such samples, carbides are not dissolved by solutioning and persist after first and second aging. A slight coarsening occurs during first aging, while second aging causes a more pronounced phenomenon. Main morphological data obtained from image analysis are reported in Table 3. Carbides formed within grains and at grain boundaries are characterized by different shape, being the latter more elongated and irregular in shape. Independently by their nucleation site, all carbides have a similar chemical composition (Figure 11) and are rich in Mo. According to their morphology and Mo content, they can be classified as M_6_C type [31,32,37,43]

**Figure 12:**
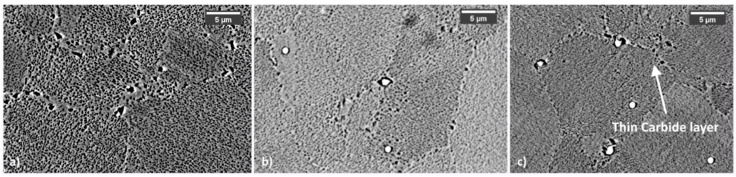
HT samples after solutioning (**a**), after first aging (**b**) and after second aging (**c**).

Carbides evolution in HT samples is very similar to that recorded in LT ones, but, as it can be seen in Figure 12, they appear smaller after solutioning. This is a direct consequence of the higher solutioning temperature applied. Main data collected from carbides analysis are reported in Table 4. By contrast with LT samples, during the second aging together with Mo carbides Cr carbide also forms at grain boundaries generating in some rare occasions a semi-continuous and very thin film. This effect is typically not positive as it can provide brittleness to the material. According to their preferential location and chemical composition, such carbides can be identified as M_23_C_6_ [31,32,37,43].

**Figure 13:**
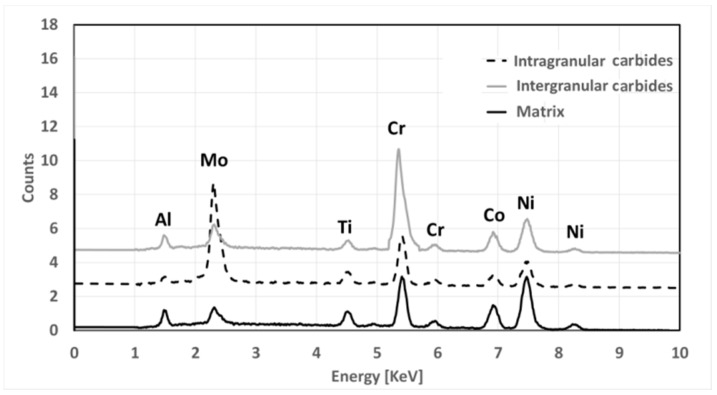
EDS spectra comparing the matrix (solid black line), Mo carbides inside a grain (black dotted line) and Cr carbides at the grain boundary (solid gray line).

In Figure 13, EDS spectra show the chemical composition of carbides relative to their location.

### 3.6. X-ray Diffraction (XRD) Analysis

**Figure 14:**
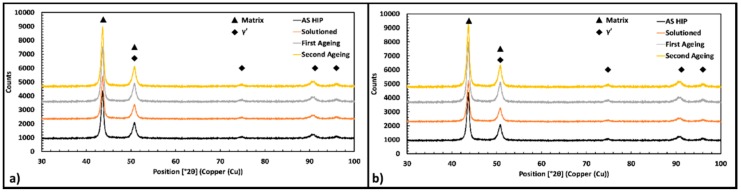
X-ray diffraction (XRD) patterns obtained from Astroloy samples in LT metallurgical condition (**a**) and in HT (**b**) respectively.

Figure 14 shows the XRD pattern obtained for Astroloy samples in all the metallurgical conditions previously described. In the provided diffractograms the peaks referring to the austenitic matrix and γ′ are visible. On the other hand, the volume fraction of carbides is too small to be detected by the instrument, thus these phases are not indicated. More precisely, Figure 14a,b show slight variation in the peaks referring to the γ′ phase. The two ageing treatments promote the precipitation of the reinforcing particles, thus the slight modification of these peaks can be related to this phenomenon. As can be imagined, the height of the peaks referring to the γ′ phase is reduced in the solutioned conditions.

## 4. Discussion

### 4.1. γ′ Reinforcing System

Independently from the temperature used during solutioning treatment, major hardness increase derives from first aging. Second low-temperature aging slightly modifies the microstructure and final hardness. In LT samples, despite a lower γ′ dissolution, the effect of hardness increase is more pronounced upon first aging. This effect can be related to the nucleation of “ultra-fine” $\gamma_{t}^{\prime }$ which is characterized by an average size of 50 nm that effectively strengthens the matrix. The following slight increase in hardness detected during sethe cond ageing could be related to the increase of $\gamma_{t}^{\prime }$ amount which, although bigger than ultrafine one, provides a certain reinforcement. This effect can recover the progressive loss of ultra-fine $\gamma_{t}^{\prime }$ which passes from 6 to 3%. The improvement in hardness recorded in HT samples with respect to LT samples, can be ascribed to the more efficient solutioning of coarse $\gamma_{p}^{\prime }$ generated by HIP consolidation. Actually, the solutioning of such precipitates releases a high amount of γ′ forming elements into the matrix. This availability of these elements favors the intense precipitation of fresh fine cuboidal γ′, that results in an effective reinforcement.

This process reaches its maximum intensity after the first aging when other $\gamma_{s}^{\prime }$ and $\gamma_{t}^{\prime }$ are formed. Precipitation is concentrated within the first ageing step while this phenomenon is less prominent during the second aging, this explains the small hardness increase obtained.

Evolution of γ′ precipitates derives from different mechanisms, but the root reason is a competitive coarsening which leads to the reduction of the interface between γ and γ′ or in other words, an Ostwald ripening process [44,45,46,47]. It was observed that $\gamma_{s}^{\prime }$ and $\gamma_{t}^{\prime }$ coarsening, occurring during first and second ageing, can be described by the following equation as suggested by [48]:

(1) $${\left(l_{t}-l_{0}\right)}^{3}=kt$$

Depending on which category of γ′ intermetallics are concerned, l_t_ is the average edge length of either $\gamma_{s}^{\prime }$ or $\gamma_{t}^{\prime }$ after a certain ageing time. Similarly, l_0_ is the average size of $\gamma_{s}^{\prime }$ or $\gamma_{t}^{\prime }$ after solutioning treatment expressed in nm as calculated in a previous work by the same authors [8]. Finally, k is a proportional constant and t is the time expressed in hours. The above equation resulted in being inadequate for $\gamma_{p}^{\prime }$ and thus this category of precipitates will not be discussed.

**Figure 15:**
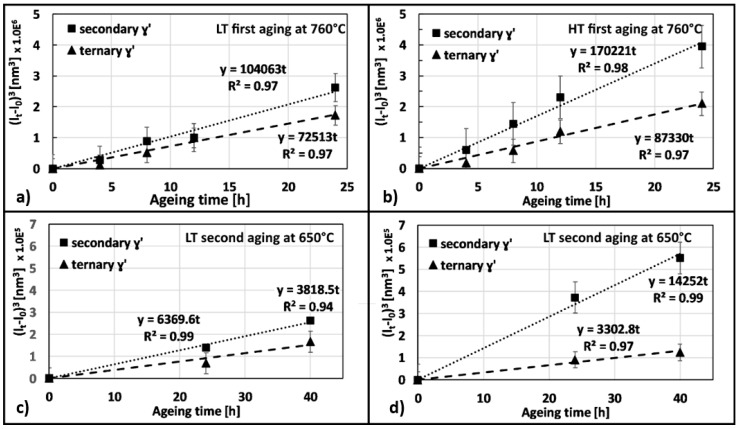
Plot of the cubic difference between the side length at a certain aging time and the side length of γ′ just after solutioning treatment. (**a**) first aging on LT, (**b**) first aging in HT, (**c**) second aging in LT and (**d**) second aging in HT.

Figure 15 shows that $\gamma_{s}^{\prime }$ grows faster than $\gamma_{t}^{\prime }$ and, in particular, that coarsening is more pronounced for HT samples. As far as the second ageing is concerned, the slope of the interpolating line describes an even slower process, nevertheless, again $\gamma_{s}^{\prime }$ grows faster than $\gamma_{t}^{\prime }$.

According to this treatment, one can easily conclude that the effect of second aging at low temperature is practically negligible for microstructure evolution and as a consequence, for properties improvement.

### 4.2. M_6_C and M_23_C_6_ Carbides

**Figure 16:**
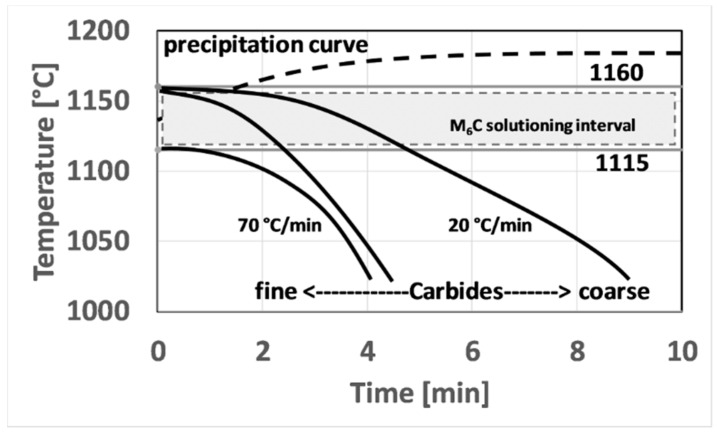
Schematic showing how solutioning temperature and cooling rate affects carbides dimension.

As far as the carbides population are concerned, analyzing the gathered data and comparing them with literature [4,5,6,7,19,20,49] one can draw the following conclusions. Within grains, only M_6_C carbides rich in Mo can form. These carbides form during HIPping at high temperature. Since cooling from HIP temperature was very slow, ca. 20 °C/min, these carbides can coarsen. As for HIP post-treatment according to literature, an 1160 °C soaking step can result in the partial dissolution of these carbides. This was experimentally verified in this study by the fact that after solutioning at 1160 °C and cooling at 70 °C/min carbides are finer. By contrast, solutioning at 1115 °C does not provide any modification to the carbides structure, since this temperature is below both γ′ and carbides solvus temperature. Major conclusions about Mo-rich carbides evolution are schematically resumed in Figure 16.

M_23_C_6_ preferentially form at grain boundaries, where a higher amount of free C can bond with Cr [4]. This kind of carbides was detected only in HT samples and after the second aging treatment. At this stage, these carbides form a very thin film surrounding γ grains. Thus, it can be affirmed that the second aging treatment for HT samples promotes the formation of such kind of carbides, structures that can act as a crack initiator. It is noteworthy to remark that M_23_C_6_ were not detected in LT samples, indicating that their formation requires a more intense Cr migration towards grain boundaries that can be achieved only through a high-temperature solutioning stage.

## 5. Conclusions

In this work, the effects of first and second aging on differently solutioned samples were studied. The major conclusions drawn are:

1. LT and HT samples benefit most from the first aging. The second aging only slightly improves material hardness. This can be explained with the fact that only minor microstructural changes occur during this treatment step. Peak hardness is achieved after first aging performed at 760 °C for 8 h.
2. LT samples achieved a greater hardness increase after first and second aging mainly due to the intense precipitation of finer γ′ i.e., $\gamma_{t}^{\prime }$. Furthermore, a “ultra-fine” $\gamma_{t}^{\prime }$ was also revealed after first ageing.
3. $\gamma_{s}^{\prime }$ and $\gamma_{t}^{\prime }$ coarsening occurs during the first and second ageing. This behavior was accurately described through a theoretical treatment evidencing that $\gamma_{s}^{\prime }$ always grows faster than $\gamma_{t}^{\prime }$. Furthermore this treatment allowed to clearly demonstrate that HT samples are more prone to coarsening.
4. M_6_C, molybdenum carbides, form directly during HIPping and can only be partially solutioned during the HT solutioning, while they remain unaltered during the LT one. M_23_C_6_ appears only in HT sample and after second aging. In this state, they form very thin films at grain boundaries which could cause material embrittlement.
5. According to the small increase in material properties and the risk of film carbides formation at grain boundaries, leads to suggestion not to perform the second aging on HIP consolidated Astroloy, in particular, if high-temperature solutioning is applied. The suppression of the second aging step is also very attractive from a practical and industrial point of view as it can result in marked savings both in terms of processing time and cost.

## Figures and Tables

**Table 1:** Alloy chemical composition.

Element	Ni	Co	Cr	Mo	Al	Ti	Fe	Zr	N	C	S	O
w%	bal	17.8	14.3	5.62	4.6	3.68	0.18	0.05	0.004	0.014	<0.002	0.01

**Table 2:** List of solutioning pre-treatment.

Sample Name	Solutioning Temperature [°C]	Cooling Rate after Solutioning [°C/min]	γ′ Fraction [% vol]
LT	1115	70	44
HT	1160	70	35

**Table 3:** Evolution of fraction of area occupied by carbides and their average diameter in LT samples from solutioning to second aging.

Condition	Fraction of Area [%]	Average Diameter [nm]
**Solutioned**	0.35	630 ± 45
**First ageing**	0.37	637 ± 61
**Second ageing**	0.40	659 ± 53

**Table 4:** Evolution of fraction of area occupied by carbides and their average diameter in HT samples from solutioning to second aging.

Condition	Fraction of Area [%]	Average Diameter [nm]
**Solutioned**	0.23	321 ± 82
**First ageing**	0.30	530 ± 53
**Second ageing**	0.35	580 ± 78
