# Correction: Bassini, E.; et al. Study of the Effects of Aging Treatment on Astroloy Processed via Hot Isostatic Pressing. *Materials* 2019, *12*, 1517

**DOI:** 10.3390/ma13081831

**Published:** 2020-04-13

**Authors:** Emilio Bassini, Giulio Cattano, Giulio Marchese, Sara Biamino, Daniele Ugues, Mariangela Lombardi, Gianfranco Vallillo, Benjamin Picqué

**Affiliations:** 1 Politecnico di Torino, DISAT: Department of Applied Science and Technology, Corso Duca degli Abruzzi, 24, 10129 Torino, Italy; giulio.marchese@polito.it (G.M.); sara.biamino@polito.it (S.B.); daniele.ugues@polito.it (D.U.); Mariangela.lombardi@polito.it (M.L.); 2 Center for Sustainable Futures @ Polito, Istituto Italiano di Tecnologia, Corso Trento 21, 10129 Turin, Italy; giulio.cattano@polito.it; 3 Consorzio Interuniversitario Nazionale per la Scienza e Tecnologia dei Materiali (INSTM), Via G. Giusti, 9, 50121 Firenze, Italy; 4 Avio Aero, Engineering Materials and Processes, Via I Maggio, 99 10040 Rivalta di Torino (TO), Italy; Gianfranco.Vallillo@avioaero.it; 5 AUBERT & DUVAL–Pamiers Business & Development manager NNS & NS Parts Powder Metallurgy 75, bd de la Libération-B.P. 173 09102 Pamiers CEDEX, France; benjamin.picque@eramet-aubertduval.com

The authors wish to make the following correction to the paper [1]. Due to a copyright issue in Figure 1, replace:

**Figure 1.** Astroloy in the as-HIPped (hot isostatic pressing) condition **a**); after sub-solvus solutioning **b**); after super-solvus solutioning **c**).

With

**Figure 1.** Astroloy in the as-HIPped (hot isostatic pressing) condition (adapted from [8]); a) after sub-solvus solutioning b); after super-solvus solutioning c).

These changes have no material impact on the discussion and conclusions of the paper. The authors would like to apologize for any inconvenience caused to the readers by these changes.
